# Predictive Power Estimation Algorithm (PPEA) - A New Algorithm to Reduce Overfitting for Genomic Biomarker Discovery

**DOI:** 10.1371/journal.pone.0024233

**Published:** 2011-09-15

**Authors:** Jiangang Liu, Robert A. Jolly, Aaron T. Smith, George H. Searfoss, Keith M. Goldstein, Vladimir N. Uversky, Keith Dunker, Shuyu Li, Craig E. Thomas, Tao Wei

**Affiliations:** 1 Translational Science, Lilly Research Laboratories, a Division of Eli Lilly & Co., Indianapolis, Indiana, United States of America; 2 Toxicology, Lilly Research Laboratories, a Division of Eli Lilly & Co., Indianapolis, Indiana, United States of America; 3 School of Informatics, Indiana University-Purdue University Indianapolis, Indianapolis, Indiana, United States of America; 4 Center for Computational Biology and Bioinformatics, School of Medicine, Indiana University, Indianapolis, Indiana, United States of America; 5 Department of Molecular Medicine, University of South Florida, Tampa, Florida, United States of America; 6 Institute for Biological Instrumentation, Russian Academy of Sciences, Pushchino, Moscow Region, Russia; Semmelweis University, Hungary

## Abstract

Toxicogenomics promises to aid in predicting adverse effects, understanding the mechanisms of drug action or toxicity, and uncovering unexpected or secondary pharmacology. However, modeling adverse effects using high dimensional and high noise genomic data is prone to over-fitting. Models constructed from such data sets often consist of a large number of genes with no obvious functional relevance to the biological effect the model intends to predict that can make it challenging to interpret the modeling results. To address these issues, we developed a novel algorithm, Predictive Power Estimation Algorithm (PPEA), which estimates the predictive power of each individual transcript through an iterative two-way bootstrapping procedure. By repeatedly enforcing that the sample number is larger than the transcript number, in each iteration of modeling and testing, PPEA reduces the potential risk of overfitting. We show with three different cases studies that: (1) PPEA can quickly derive a reliable rank order of predictive power of individual transcripts in a relatively small number of iterations, (2) the top ranked transcripts tend to be functionally related to the phenotype they are intended to predict, (3) using only the most predictive top ranked transcripts greatly facilitates development of multiplex assay such as qRT-PCR as a biomarker, and (4) more importantly, we were able to demonstrate that a small number of genes identified from the top-ranked transcripts are highly predictive of phenotype as their expression changes distinguished adverse from nonadverse effects of compounds in completely independent tests. Thus, we believe that the PPEA model effectively addresses the over-fitting problem and can be used to facilitate genomic biomarker discovery for predictive toxicology and drug responses.

## Introduction

Many preclinical candidate compounds do not achieve regulatory approval because of organ toxicity and lack of adequate safety margins. Up to half of these compounds are terminated from development due to hepatotoxic effects including necrosis, steatosis, cholestasis, proliferation, inflammation, and bile duct hyperplasia (BDH) [1]. It has been well-documented that biomarkers that identify incipient damage that lead to preclinical and clinical toxicities will enable better decision-making during drug development [2]. Particularly valuable are translational biomarkers that bridge preclinical testing species and humans as they can expand the usefulness of the former for detection of human liabilities [3].

Although a sole biomarker is appealing as it can be less expensive to measure, and the results are easier to interpret than a panel of markers, there are few examples in preclinical testing or in clinical practice wherein a single measurement is considered definitive for target organ toxicity. Multiple markers are required to capture the biological heterogeneity of organs involved, individual variations and disease or toxicity processes [4]. Microarray technology allows us to observe and assess the expression of thousands of genes simultaneously in each sample and machine learning algorithms can be applied to identify gene signatures or biomarkers from microarray data. Numerous recent studies have demonstrated that gene expression signatures not only outperform traditionally used clinical parameters in toxicity or disease outcome prediction, but also contribute to a better understanding of the biological mechanism [5]–[10]. However, gene signatures proposed to be correlated to the same biological phenotype by different researchers differ widely and often have very few genes in common [11]–[12]. This lack of congruence raises doubts about the reliability and robustness of the reported predictive signatures. Analysis of the situation has led to suggestions that the different gene sets may result, at least in part, from over-fitting [13]–[15]. Over-fitting is a concern when the number of training samples is small and the number of genes relatively large, as in this situation it is straightforward to obtain a classifier that correctly describes the training data, but performs poorly on an independent set of data.

Over-fitting has been closely examined in several studies [16]–[17]. Two studies in logistic and Cox regression showed increasing bias and variability, unreliable confidence interval coverage, and problems with model convergence as events per variable (EPV) declined below 10, and especially below five, leading to the rule of thumb that logistic and Cox models should be used with a minimum of 10 EPV [18]–[19]. Therefore, feature selection is commonly performed before sample classification is even attempted to alleviate the above stated problem. Although numerous reports for feature selection have been published, and some techniques have been claimed better than others [13], [20]–[22], to date, no single recommendation in the literature is given for methods in either the feature selection or the classification of microarray data [22]-[23].

Feature selection algorithms fall mainly into two broad categories, the filter model or the wrapper model [24]–[26]. The widely accepted filter techniques are single-feature based and have been demonstrated to be effective for improving sample classification accuracy. Some of them are statistical tests (t-test, F-test) [27], non-parametric tests like TNoM [28], S2N ratio (signal to noise ratio) [29], etc. However, these methods share a limitation in that the interaction with classifier and feature dependencies has been completely ignored. However, interactions between genes are important for numerous - if not all - biological functions [30]–[31]. Although the *wrapper* methods use the interactions between features, perform multivariate gene subset selection, and incorporate the classifier's preference or bias into the search and thus offer an opportunity to construct more accurate classifiers, the disadvantages are that they are computationally intensive, result in classifier dependent selection, and are at a particularly high risk of over-fitting [22]. In the present study, we describe the development of a new method, Predictive Power Estimation Algorithm (PPEA), to evaluate and rank the relative predictive power of individual genes. By applying PPEA to the DrugMatrix™ chemogenomic database, we identified and validated three small sets of genes highly predictive of, and functionally related to, liver inflammation (INFL), necrosis and bile duct hyperplasia (BDH), respectively. Furthermore, we successfully converted a 3-gene signature to a multiplex qRT-PCR assaythat can be effectively deployed as a genomic biomarker to predict BDH.

## Materials and Methods

### Ethics Statement

All animals were handled in strict accordance with good animal practice as defined by the relevant national and local animal welfare bodies, and all animal work was approved by the Lilly's Institutional Animal Care and Use Committee under IACUC protocol 2008-0727.

### Algorithm

**Figure 1:**
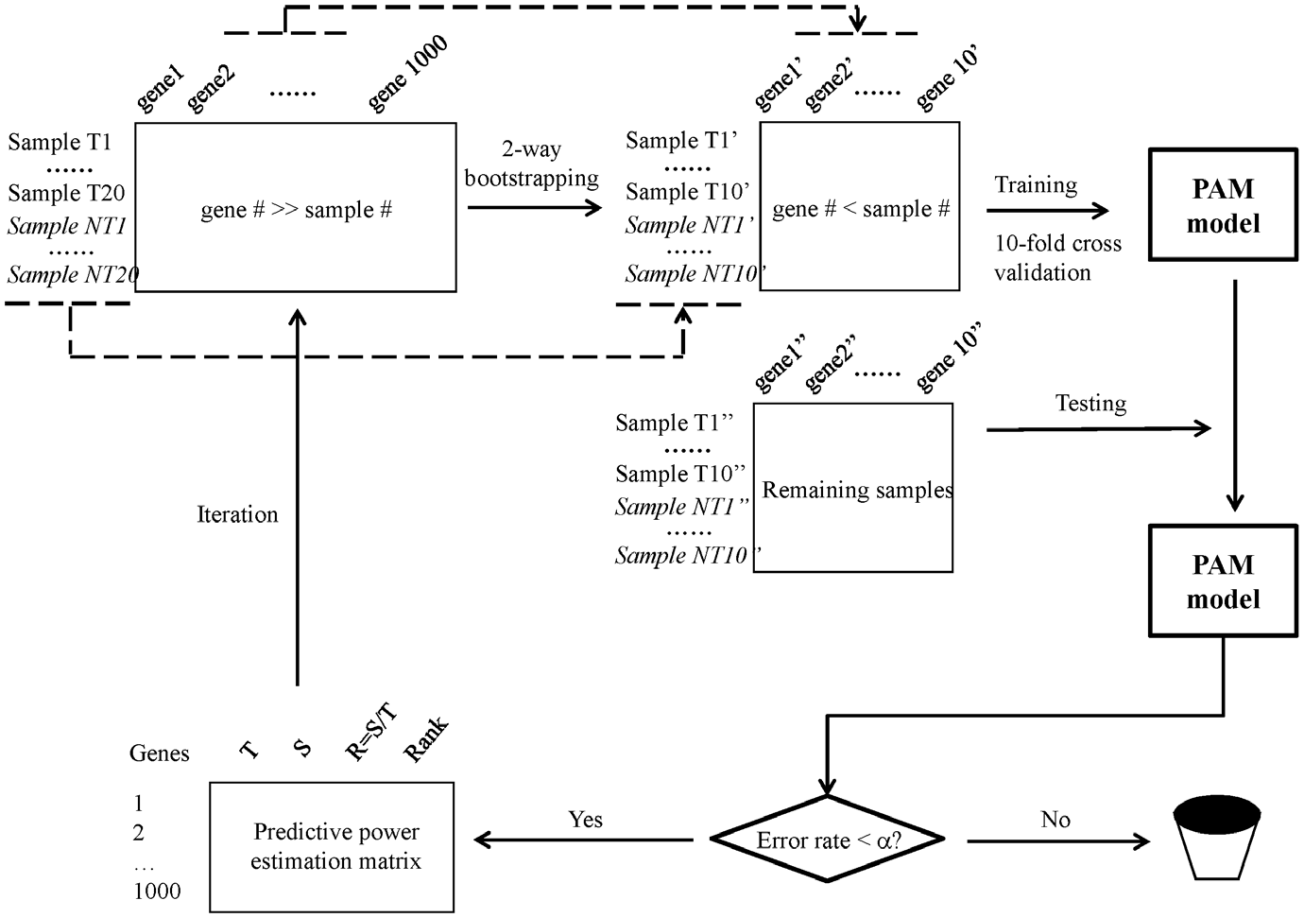
Schematic representation of PPEA algorithm. Assumptions used in the schema are (1) the original data matrix consists of (20 toxic and 20 non-toxic samples) x1000 genes, and (2) a 2-way bootstrapping sample consists of 10 toxic and non-toxic samples (i.e. ½ splitting ratio) and 10 genes.

Figure 1 shows the PPEA algorithm. Let 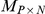 be the expression data matrix of *P* genes as rows and *N* samples as columns, among which 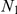 samples are labeled as 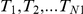
for toxicity class and 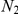 samples labeled as 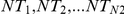 for non-toxicity class. Thus, 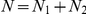. Let *α* be a predetermined threshold of acceptable classification error rate of model testing and β be the arbitrarily defined sample split ratio to construct training and testing sample sets. Let K be the total number of iterations and *k* be the 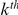 iteration 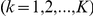. Let 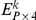
be the performance matrix estimated after the 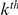 iteration consisting of *P* rows, each of which is identified by the genes 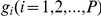
in the data matrix 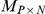, and four columns corresponding respectively to 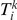
as the total number of times 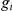 is sampled after 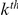 iterations, 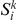 as the total number of times 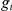 selected in the successful predictive modeling (see Step 3a below for definition) after 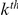 iterations, 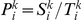 as an estimate of the predictive power of 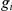 after the 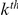 iterations, and 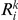 *where* 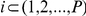 as estimated rank order of 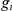 based on its estimated predictive power 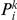
after the 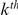
iteration. Genes with larger 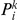 are more predictive than those with smaller 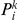
and thus ranked higher. At the initiation of the algorithm, 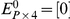. For each iteration 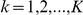, executes the following steps.

**Step 1:** Apply two-way bootstrapping to the 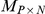 to obtain a bootstrapping sample matrix 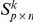 consisting of *p* genes, 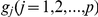, randomly drawn from *P* genes, 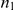 samples from 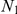 samples of toxicity class and 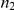 samples from 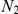 samples of non-toxicity class such that 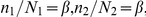 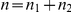 and 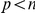. 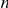 is the sample size of training sample set while 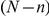 is the sample size of testing sample set.

**Step 2:** Apply Prediction Analysis of Microarray (PAM) to the bootstrapping sample matrix 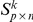
to perform sample classification using the nearest shrunken centroid method [32]. To build a predictive PAM model, ten-fold cross validation was performed to determine the optimal classifier performance which minimizes classification errors for the training set 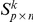 Based on the ten-fold cross validation, a threshold 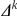 was varied in search of the optimal classifier performance. The 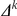 is chosen when the lowest classification errors achieved with the fewest genes 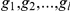 *where* 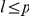. The resultant PAM model in the current 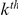 iteration
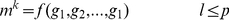
(1)is subsequently tested using the 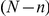 testing samples. Let *e* be the error rate of the 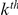 modeling when tested with the testing samples and estimated by (2). 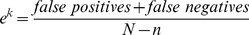
(2)


In cases where cross validation errors are greater than 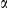 for all possible 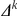 value, i.e., no acceptable PAM model can be constructed from genes 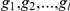 *where* 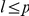 for the training samples, the independent model test using 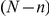 testing samples described above is omitted and the execution proceeds to Step 3b described below.

**Step 3a:** If 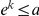, *i.e.,* the estimated error rate of the model tested with 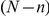 testing samples is less than the predetermined threshold, the model is deemed to be predictive and thus a successful modeling. The performance matrix 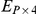 is updated as follows. Each gene, 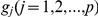, in the bootstrapping samples 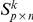
is mapped to 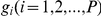 in 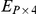, 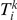, 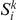, and 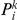 are updated sequentially as follows:
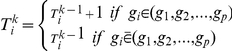


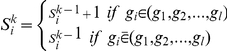


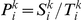



**Step 3b:** On the contrary, if 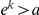, *i.e.,* the estimated error rate of the model tested with 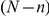 samples is larger than the predefined threshold, the model is deemed to be not predictive for independent testing samples. The model constructed is over-fitting and thus is discarded. 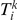, 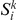, and 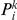 in the performance matrix 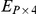 are updated sequentially as follows:
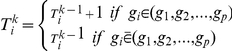


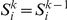


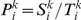



Sort 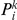 decreasingly, *i.e.,* 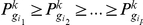, a rank order of genes in term of their predictive power is given as
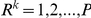



#### Stop criterion

The rank order 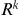 is evaluated periodically, say every 10000 iterations, by computing Spearman correlation coefficient between the current rank 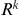 and the previous rank 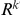, which is the previous rank order after the 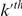 iterations, *i.e.,*

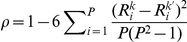



The algorithm stops if 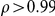, *i.e.,* the iteration stops when the rank is stabilized.

### Case studies

DrugMatrix™ is a chemogenomics database originally developed by Iconix Pharmaceuticals, now owned by Entelos, Inc. [33]. In a typical DrugMatrix™ toxicology study, three rats in each combination of dose and time point (defined as a treatment) were used to generate three independent biological replicate RNA profiles using the RU1 microarray (Agilent). All data were MIAME compliant and raw data have been previously deposited to a MIAME compliant database, GEO, accession GSE8858 [34]. An average profile representing each treatment, defined as a sample, was computed from the three biological replicates. The PPEA algorithm was tested using data from DrugMatrix™ studies that resulted in three different liver toxicities, namely bile duct hyperplasia (BDH), necrosis, and inflammation (INFL). For each toxicity phenotype, two classes of compounds were identified by querying the DrugMatrix™ database based on severity and incidence of the phenotype they induced. A compound was classified into the positive class if its p-value of observed idit score [35]–[36] for a given phenotype is less than or equal to 0.01 and the percentage of incidence is 100. The ridit analysis is a statistical technique that works with ordered categorical data by evaluating categories in terms of their frequency of occurrence in a control or reference group [35]–[36]. Conversely, a compound was classified into the negative class if its p-value of observed ridit score for a given phenotype is larger than 0.5 and percentage of incidence is 0. Compounds with intermediate p-values and percentage of incidences were excluded from the analysis. These high stringency criteria provided us with a reliable identification of robust positive and negative classes of samples for modeling. Note that a single compound may contribute multiple samples for analysis owing to different time and dose combinations which pass the query criteria. The number of compounds and treatments in the positive class for each phenotype are listed in Supporting Information Table S1.

The Uniset Rat I Expression (RU1) microarrays used for the experiments described here were purchased from Amersham Biosciences (Piscataway, NJ, now part of GE Healthcare, USA). The RU1 BioArray contains 9911 nucleotides probes (30-mer) with 8565 probes used for data analysis. Previous studies [34], [37] have shown that low-abundant transcripts tend to be much more variable than abundant ones. Thus, three filters were applied to obtain informative transcripts for the modeling process. An informative transcript is defined as one with: (1) average expression larger than 2 on a 1 to 5 scale defined in DrugMatrix™, (2) absolute fold changes larger than or equal to 1.5, and (3) a p-value less than 0.05 when the treatment group is compared with the vehicle using Student t-test. Using these criteria, 4231 informative transcripts were identified.

### Pathway analysis

The most predictive, i.e., top-ranked 20 genes, from each case study were analyzed using Ingenuity Pathway Analysis tool (purchased from Ingenuity Systems, Redwood, CA) to assess their potential functional relevance with the liver toxicity phenotype which the gene set was intended to predict.

### Generation of predictive model from the top-ranked genes by PPEA

PPEA was implemented in the R release 2.9 (http://www.r-project.org/, the R code is provided as Supporting Information Text S1). For each case study, the top-ranked 10 genes with all samples were used as an initial input to train PAM models. The smallest model was obtained with an arbitrarily pre-determined acceptable error rate, i.e. ≤20%, based on the ten-fold cross-validation. In cases that such a model did not exist due to unacceptably high error rates, i.e. >20%, expanded top-ranked genes such as 15, 20 or more may be attempted. For all cases in the present study, we found the top 20 genes were sufficient to obtain a predictive model for each toxicity phenotype with acceptable error rates based on the 10-fold cross validation. The resultant model obtained in this way is called the PPEA-PAM model to distinguish it from the PAM model without feature selection by PPEA.

For each case study, we compared our model with the best PAM model without applying PPEA as well as with the respective gene signatures of DrugMatrix™, which were developed by sparse-SVM [33]–[34]. Sensitivity, specificity, positive and negative predictive values (PPV and NPV respectively) of the PPEA-PAM model were compared with those of PAM and DrugMatrix™ gene signatures.

### Quantitative reverse transcription-polymerase chain reaction (qRT-PCR)

Total RNA was isolated from banked frozen rat livers from the same studies used to generate DrugMatrix™ RNA profiles by placing ∼0.3 mg of liver in 800 µl of RLT lysis buffer (RNeasy minikit Qiagen) in Lysing D matrix tubes and homogenized using a Fastprep FP-120 tissue homogenizer (Bio101 Systems). Based on their expression difference between positive and negative classes in the microarray study, three genes (RhoC, Jub, and Pspla1) were selected from our PPEA-PAM model for BDH for validation by quantitative real-time RT-PCR (qPCR). A housekeeping gene peptidylpropyl isomerase b (Ppib), also known as cyclophilin, is used as a control gene because this gene is reported as an endogenous reference for gene expression analysis [38]-[39]. Primers were designed with Primer Express software and purchased from Applied Biosystems (Palo Alto, CA, USA). For qPCR, 1 µg total RNA was reverse transcribed in a final volume of 50 µl using high capacity cDNA reverse transcription reagents (Applied Biosystems) with random hexamer primers according to manufacturer instructions. Reactions excluding MultiScribe Reverse Transcriptase (Applied Biosystems) were performed as negative controls. cDNA targets at a 50-fold final dilution were amplified in three replicate wells in an ABI 7900 Sequence Detector System (Applied Biosystems) with the following thermal profile: 50°C for 2 min, 95°C for 10 min, followed by 40 cycles of 15 sec at 95°C and 1 min at 60°C. Standard curves for each gene were obtained by amplifying (in triplicates) 5-fold serial dilutions of a reference mixture containing cDNA derived from treated and control tissues.

### Confirmation of predictive value of the top-ranked genes by PPEA

To independently validate the predictive value of the top-ranked genes by PPEA, for each case study, we selected compounds originating from our internal drug development programs which were terminated due to observed rat liver toxicity (i.e. BDH, INFL or Necrosis). Compounds which did not display the indicated toxicity phenotype were also identified and served as negative controls. Total RNA was prepared from rat livers of selected animals and approximately 5 µg of total RNA was reversed transcribed into cDNA using a Superscript II Double-Strand cDNA Synthesis Kit (Invitrogen Life Technologies). 5µg of which was used to hybridize Affymetrix rat genome DNA hip RAE2302 430A 2.0, which contains sequences corresponding to roughly 22,600 transcripts, according to Affymetrix protocol (http://www.affymetrix.com/support/technical/manuals.affx). AnAffymetrix fluidics station 400 was used for array washing and staining, and an Agilent GeneArray scanner 3000 was for scanning. Each sample was hybridized to a single microarray. Expression of selected genes for each case study was obtained from the DNA chips and analyzed using principal component analysis (PCA), and also served as an independent test set for the model assessment with Support Vector Machine (SVM). The sensitivity, specificity, and accuracy were calculated.

## Results

### Estimation of predictive power of individual transcripts by PPEA

**Figure 2:**
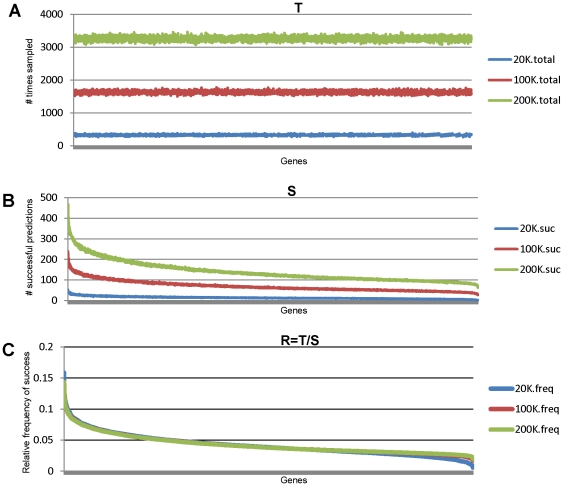
Analysis of sampling distribution in the predictive power enrichment matrix. (**A**) A random number generator with a uniform distribution was used so that each of 4000 features (genes) had equal chances to be sampled. Y axis is the total # of times a gene was sampled, represented as T. (**B**) A prediction was called a success if overall error rates < α, which is 20%. Y axis is the total # of times a gene was included in a successful modeling, denoted as S. (**C**) Y axis is the Relative Success Rate that a gene was used in successful modeling, computed as R = S/T, which is a metric to measure the predictive power of the gene.

Two-way re-sampling with replacement or bootstrapping was applied to the data matrix iteratively as described above in the algorithm section. As shown in Figure 2(A), all transcripts had an equal chance to be evaluated for its predictive power by the PPEA algorithm, as expected by design. The total number of evaluations for each transcript is proportional to the total number of iterations executed. For example, on average each transcript was evaluated 300 times for a total of 20,000 iterations, which increased to 1500 and 3000 times respectively for a total of 100,000 and 200,000 iterations. Different transcripts have different predictive power, as shown in Figure 2(B), when measured by the total number of successful modeling iterations, i.e. their error rates computed from the testing samples are less than an arbitrarily determined threshold 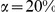. The differences in successful product was small (compare the left and the right ends of the blue plot of Figure 2(B)) when the total number of iterations was low, for example 20,000. Then the differences increased and became obvious as the total number of iterations increased, as shown by magenta and pink plots in Figure 2(B). Such dependency on the total number of iterations disappeared when normalized to the total number of times each transcript was evaluated (Figure 2(C)). Thus, PPEA can quantify the predictive power of a large number of transcripts.

**Figure 3:**
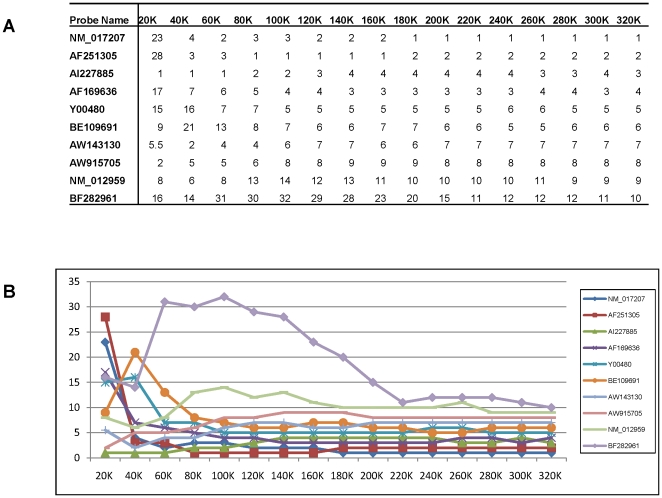
Example of top 10 genes Rank shifting at each checkpoint of the iteration. (**A**) shows that the index of the 10 top-ranked genes (i.e., features) becomes stabilized when the iteration of splitting reached 280k. The rank for 8 out of 10 genes is consistent as early as the iteration reaches to 180k. (**B**) A plot for the same data as shown in (**A**) for an intuitive observation.

In principle, PPEA has to evaluate all 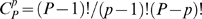 possible combinations of *p* out of *P* transcripts, which is an 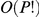 algorithm, in order to estimate the true predictive power of a transcript. Thus, it is of great interest to determine if a relative rank order of predictive power of transcripts can be reliably obtained by the bootstrapping procedure. Using BDH as an example, we first executed 320,000 iterations of PPEA to obtain a distribution of predictive power of all transcripts and then obtained a rank order based on their estimated predictive power sorted in decreasing order. We compared the rank orders similarly obtained from a smaller number of iterations. As shown in Figure 3A, the rank order of predictive power for BDH among the top ten transcripts were very different for the 20K and 40K iterations. However, the rank order of the top 10 genes was largely stabilized after 180K iterations without significant changes (Figure 3B). Similar results were obtained for INFL and necrosis. Thus, PPEA can reliably obtain a relative rank order of the most predictive transcripts using a relatively small number of iterations.

### Assessment of the top ranked transcripts

**Figure 4:**
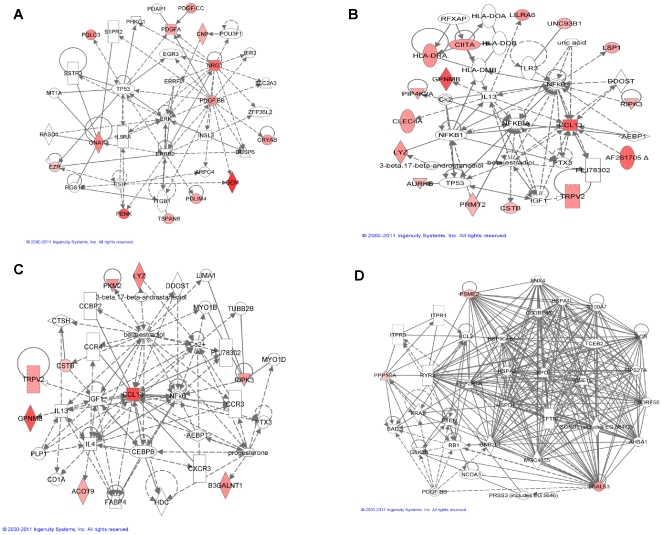
Ingenuity Pathway Analysis (IPA) for the enriched biological functions of the top 20 genes for each signature. The involved genes are highlighted in the red color. The lines between genes represent known interactions, with solid lines representing direct interactions and dashed lines representing indirect interactions. The high scores associated with these networks indicate they were highly unlikely to be formed by chance. (**A**) 11 out of the top 20 genes for BDH signature are involved with p53 and ERBB2 pathways (Network 1, Cancer, Cell Cycle, Cell Death, score of 28), and (**B**) 17 out of the top 20 inflammation signature genes are involved with inflammatory pathway NFκB (Network 1, Immune Response, Cell-To-Cell Signaling and Interaction, Connective Tissue Disorders, score of 47). (**C**) 9 of the top 20 genes for the necrosis signature are also involved with Immune Response pathway, majorly NFκB complex, and (**D**) 3 of the top 20 genes for necrosis are associated with cell death (Network 2, Cancer, Cell Death, Cell Cycle, score of 6).

Most of the reported gene signatures derived purely mathematically consist of many genes with either no functional annotation (*e.g*. EST's) or no obvious functional relevance to the phenotype they predict [11]–[12]. We believe this may, at least partially, originate from overfitting. To assess if PPEA selects genes that were associated or correlated to the toxicity phenotype, we performed pathway analysis of the twenty top ranked genes for BDH, Necrosis, and INFL (see Supporting Information Table S2 for detail) using Ingenuity Pathway Analysis tools (Ingenuity® Systems, www.ingenuity.com). As shown in Figure 4A for BDH, eleven out of the twenty top ranked genes participate in the cell proliferation interaction network. They have either direct or indirect functional interactions with powerful mitogenic factors such as ERBB2, a well-known member of the epidermal growth factor (EGF) receptor family of receptor tyrosine kinases. Amplification and/or over-expression of this gene has been reported in numerous cancers, including breast and ovarian tumors [40]–[41]. Similarly, as shown in Figure 4B for INFL, seventeen out of the twenty top ranked genes were mapped to the inflammation and immune response network where they directly or indirectly interact with the key inflammatory regulator NFκB [42]. Interestingly, the highest scoring network represented within the top twenty ranked genes for necrosis related to immune response, linking 9 necrosis-associated genes to the well defined inflammatory NFκB pathway (Figure 4C). A second network links 3 members of these top 20 necrosis genes to cell death and cell cycle (Figure 4D). This result implies that liver necrosis is strongly associated with, or resulted in, the hepatic inflammation, which is a common finding with liver damage, including drug-induced liver toxicity [43]. Importantly, about one third (14) of the positive compounds in this study caused both inflammation and necrosis at a different time period and/or dose level than those that reported just necrosis. Thus, it is clear that the most predictive genes identified by PPEA tend to be functionally related to the phenotype they predict, which is generally not true for gene signatures derived by other methods.

Current gene signatures for toxicity originally developed by Iconix Inc. using sparse-linear regression or SVM are highly predictive of phenotype [33], [44]. However, they typically consist of dozens or even hundreds of transcripts. Because of the bulky size of the gene signatures, it would be difficult for users to develop alternative assay formats, for example multiplex qRT-PCR, to facilitate fast compound screening in the early stages of drug development. Considering the plate size, number of replicates and throughput, an assay multiplexed with five to ten genes is desirable in practice. Rank order of predictive power of individual transcripts obtained by applying PPEA aids in selecting a small number of transcripts. The question is whether the top ranked small number of genes can still effectively predict toxicity phenotype. To answer this question, we first developed the minimum signature, called PPEA-PAM, for the liver toxicity phenotypes of BDH, necrosis, and INFL, and progressively selected from 10, 15 or 20 top-ranked transcripts. Next, we compared performance of PPEA-PAM with that of PAM and corresponding DrugMatrix™ gene signatures derived from the full set of transcripts. As shown in Table 1, PPEA-PAM was comparable to, or significantly outperformed PAM and DrugMatrix™ gene signatures, in terms of overall error rates, sensitivities and specificities.

**Table 1:** A comparison of the performance of 3 different signatures for detecting Necrosis, Bile Duct Hyperplasia, and Inflammation.

			Rate (%)			P-value *	
Performance	Signature	Necrosis	INFL	BDH	Necrosis	INFL	BDH
Sensitivity	PPEA-PAM	85.10%	84.40%	95.30%	NA	NA	NA
	PAM	84.60%	82.00%	91.30%	1.59E-01	2.58E-14	1.94E-23
	ICONIX	81.70%	80.50%	93.40%	2.17E-18	1.32E-32	3.55E-07
Specificity	PPEA-PAM	75.90%	75.50%	94.50%	NA	NA	NA
	PAM	75.10%	72.20%	90.90%	9.12E-04	9.05E-44	1.11E-20
	ICONIX	73.90%	69.60%	88.10%	1.39E-12	1.5E-120	2.09E-57
Error rate	PPEA-PAM	20.50%	20.90%	5.50%	NA	NA	NA
	PAM	21.20%	23.90%	9.50%	1.03E-02	6.00E-34	3.98E-30
	ICONIX	23.00%	26.30%	10.00%	5.44E-17	1.19E-92	1.56E-38

*P value is generated from student t – test when comparing the percentage of sensitivity, specificity, and error rate for PPEA-PAM with that for PAM or ICONIX signature.

**Figure 5:**
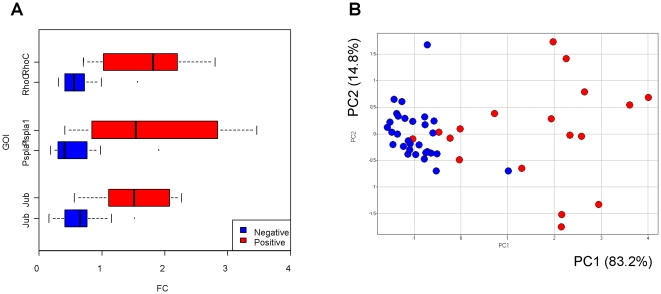
qPCR. (**A**) Boxplots represent normalized mRNA expression of the top three genes for BDH signature determined by RT-qPCR. The box represents the 25th and 75th percentile range of scores. A vertical line in each box represents the median value of the normalized mRNA of each group. The whiskers represent the highest and lowest values. The box highlighted with red and blue colors indicates the group of animals treated with BDH positive (n = 18) or negative compounds (n = 30), respectively. (**B**) Principal components analysis (PCA) shows that there is a clear separation between the positive and negative classes based on the expression level of top 3 genes in BDH signature. GOI  =  Gene of Interest.

Early prediction of BDH is challenging in short duration toxicology studies when reliant solely on histology and clinical chemistry as true hyperplasia may require a longer timeframe to manifest, thus better predictive methods are needed. We validated the expression of the three genes in the PPEA-PAM gene signature for BDH using qRT-PCR. As shown in Figure 5, positive BDH samples could be clearly distinguished from the negative samples across all samples used in signature derivation simply using the three gene signature.

### Independent confirmation of selected genes

**Figure 6:**
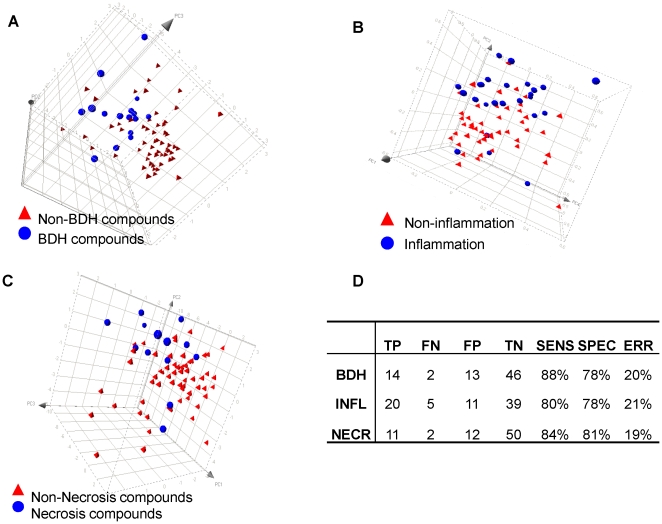
Validation with an independent dataset. Visual representation of PCA results for the top 10 genes in the BDH (**A**), INFL (**B**), and necrosis (**C**) signatures validated with independent samples. The result shows a clear separation between positive and negative compounds; (**D**) a SVM classifier performance matrix shows that a reasonable sensitivity (from 80% to 88%) and specificity (from 78% to 81%) have been achieved for each model. TP - *True Positive*; FN - *False Negative*; FP - *False Positive*; TN - *True Negative*; SENS - *Sensitivity*; SPEC - *Specificity*; ERR - *Error rate*; NECR - *Necrosis*.

To independently confirm that the genes selected by PPEA-PAM using DrugMatrix™ data are predictive for BDH, Necrosis, and INFL, we identified compounds originating from our drug development programs that were terminated due to observed adverse findings in liver (i.e. BDH, Necrosis, or INFL). Compounds which did not display the indicated toxicity phenotype were also identified and served as negative controls. Liver expression profiles of rat treated with these compounds were generated using Affymetrix DNA chips. Expression of the top 10 genes predictive for BDH, INFL, and necrosis were analyzed using PCA. As shown in Figure 6A, 6B, and 6C, BDH-, Necrosis-, and INFL- inducing compounds can be clearly separated from non-inducing compounds by expression changes of a small number of highly predictive genes. The detail information for each model performance is listed in Figure 6D.

## Discussion

A recurring question when working with microarray data is how to manage the ubiquitous “overfitting” in gene expression profiling. Because of the uniqueness of microarray data, wherein the sample size is typically far smaller than the feature size, this situation necessitates dimensionality reduction through gene selection to avoid overfitting and improve the generalization of discriminant analysis. In this paper, we propose a novel feature selection algorithm termed PPEA to tackle this fundamental issue. PPEA first applies two-way bootstrapping to manage the number of genes inversely equal to or less than the number of samples in each splitting subset used for machine learning, and then assesses the merit of each individual feature by evaluating its strength of class predictability under this new low dimensional sample-feature space. This approach is different from the other feature selection algorithms in that it assesses predictive power of individual genes within the context of a multivariate model. Accordingly, this enables PPEA to access the gene information contained in complex biological interactions, rather than relying on the summation of univariate relationships within a set. For example, if two genes in a category were related to the samples' biological process or state by an ‘exclusive OR’ association, then PPEA could capture that relationship, whereas filter-based summations of univariate associations would likely overlook it.

The task of conventional feature selection in microarray analysis is considered as a search problem where each state in the search specifies a distinct subset of the possible relevant features. If the search space is too large, it is possible that the algorithm cannot discover the most selective genes within the search space. Moreover, having too many redundant or irrelevant genes increases the risk of overfitting, computational complexity and cost and, ultimately, degrades estimation in classification error. The PPEA algorithm described here, in concept, approaches the search space in a “divide and conquer” fashion, breaking down the search space into a large number of sub-spaces of the same (or related) type. These sub-spaces with a new dimensionality (the sample size is now larger than the feature size) are thus able to minimize over-fitting. The solutions to the sub-space are then combined to give a solution to the original space. In practice, we realize that the random data split in each iteration may create circumstances where different runs of the algorithm may select different features if the number of iterations is small. An unfortunate split of the data set may also remove an important feature, thus negatively affecting the classifier's performance. Fortunately, this situation can be avoided if the number of iterations is large enough. We do not claim that our PPEA methods will find all interesting genes, because the schema for feature search in this algorithm is heuristic and suboptimal as it does not exhaustively search in the space of all possible combinations. However, we demonstrated that the rank transition became a plateau and that the majority of features ranked at the top positions were very stable after a certain number of iterations were searched. In our algorithm, the iteration is terminated when the stability of ordered features according to their predictive power within each predictive power enrichment matrix is reached (Figure 3).

A key and pivotal finding from the PPEA analysis is that it provides insight into the biological mechanisms for a phenotype of interest. The functional analysis demonstrates that the signature genes tend to be mechanistically related to the phenotype the signature is intended to predict, *i.e.* associated with mechanism of toxicity. For example, BDH manifests a purely cholangiolar proliferation considered as a pre-neoplastic lesion usually as a result of exposure to carcinogenic compounds such as Phomopsin [45]. Our results show that 11 of the top 20 genes for the BDH signature are associated with key regulators of cell proliferation, for example, ERBB2 (Figure 4A). We also observed that 17 out of the top 20 genes for the “INFL” and 9 of the top 20 genes for the “Necrosis” signatures identified by PPEA were members of the NFκB interactive network (Figure 4B and 4C). This finding further confirms that immune responses and associated autoimmunity can play an important role in both predictive (acute) and idiosyncratic drug-induced liver injuries [43]. We believe that our approach provides a novel method to find genes that truly reflect the biological consequences of a therapeutic intervention or disease. Furthermore, the fact that the PPEA method provides gene sets of limited number allows for the use of non-microarray methods such as qPCR which greatly reduces cost and improves on turn-around times for data generation and analysis. Herein, we have demonstrated with our liver injury datasets that quite accurate diagnoses for several distinct phenotypes could be achieved using the gene-expression level results of only 5 - 20 genes.

## Supporting Information

Text S1 The R code for PPEA algorithm.
(TXT)

Table S1 Summary of the positive class compounds and experiments which defined Bile Duct Hyperplasia, Inflammation, and Necrosis.
(XLSX)

Table S2 The list of the top 20 ranked genes for BDH, Necrosis, and INFL.
(XLSX)

## References

[1] Ozer J, Ratner M, Shaw M, Bailey W, Schomaker S (2008). The current state of serum biomarkers of hepatotoxicity.. Toxicology.

[2] Ryan TP, Stevens JL, Thomas CE (2008). Strategic applications of toxicogenomics in early drug discovery.. Curr Opin Pharmacol.

[3] Sistare FD, DeGeorge JJ (2007). Preclinical predictors of clinical safety: opportunities for improvement.. Clin Pharmacol Ther.

[4] Mendrick DL (2008). Genomic and genetic biomarkers of toxicity.. Toxicology.

[5] Fielden MR, Eynon BP, Natsoulis G, Jarnagin K, Banas D (2005). A gene expression signature that predicts the future onset of drug-induced renal tubular toxicity.. Toxicol Pathol.

[6] Luo W, Fan W, Xie H, Jing L, Ricicki E (2005). Phenotypic anchoring of global gene expression profiles induced by N-hydroxy-4-acetylaminobiphenyl and benzo[a]pyrene diol epoxide reveals correlations between expression profiles and mechanism of toxicity.. Chem Res Toxicol.

[7] Bushel PR, Heinloth AN, Li J, Huang L, Chou JW (2007). Blood gene expression signatures predict exposure levels.. Proc Natl Acad Sci U S A.

[8] Zidek N, Hellmann J, Kramer PJ, Hewitt PG (2007). Acute hepatotoxicity: a predictive model based on focused illumina microarrays.. Toxicol Sci.

[9] Eun JW, Ryu SY, Noh JH, Lee MJ, Jang JJ (2008). Discriminating the molecular basis of hepatotoxicity using the large-scale characteristic molecular signatures of toxicants by expression profiling analysis.. Toxicology.

[10] Fan X, Lobenhofer EK, Chen M, Shi W, Huang J (2010). Consistency of predictive signature genes and classifiers generated using different microarray platforms.. Pharmacogenomics J.

[11] Fan C, Oh DS, Wessels L, Weigelt B, Nuyten DS (2006). Concordance among gene-expression-based predictors for breast cancer.. N Engl J Med.

[12] Liu J, Campen A, Huang S, Peng SB, Ye X (2008). Identification of a gene signature in cell cycle pathway for breast cancer prognosis using gene expression profiling data.. BMC Med Genomics.

[13] Ransohoff DF (2004). Rules of evidence for cancer molecular-marker discovery and validation.. Nat Rev Cancer.

[14] Ransohoff DF (2005). Bias as a threat to the validity of cancer molecular-marker research.. Nat Rev Cancer.

[15] Dessì N, Pes B (2009). An Evolutionary Method for Combining Different Feature Selection Criteria in Microarray Data Classification..

[16] Sima C, Dougherty ER (2008). The Peaking Phenomenon in the Presence of Feature Selection Pattern Recognition Letters.

[17] Dougherty ER, Hua J, Sima C (2009). Performance of feature selection methods.. Curr Genomics.

[18] Vittinghoff E, McCulloch CE (2007). Relaxing the rule of ten events per variable in logistic and Cox regression.. Am J Epidemiol.

[19] Peduzzi P, Concato J, Kemper E, Holford TR, Feinstein AR (1996). A simulation study of the number of events per variable in logistic regression analysis.. J Clin Epidemiol.

[20] Guyon I, Weston J, Barnhill S, Vapnik V (2002). Gene selection for cancer classification using support vector machines.. Machine Learning.

[21] Zhang X, Lu X, Shi Q, Xu XQ, Leung HC (2006). Recursive SVM feature selection and sample classification for mass-spectrometry and microarray data.. BMC Bioinformatics.

[22] Saeys Y, Inza I, Larranaga P (2007). A review of feature selection techniques in bioinformatics.. Bioinformatics.

[23] Guyon I, Elisseeff A (2003). An introduction to variable and feature selection.. The Journal of Machine Learning Research.

[24] Das S (2001). Filters, wrappers and a boosting-based hybrid for feature selection..

[25] John GH, Kohavi R, Pfleger K (1994). Irrelevant Features and the Subset Selection Problem..

[26] Kohavi R, John GH (1997). Wrappers for feature subset selection.. Artificial Intelligence.

[27] Bo T, Jonassen I (2002). New feature subset selection procedures for classification of expression profiles.. Genome Biol.

[28] Ben-Dor A, Bruhn L, Friedman N, Nachman I, Schummer M (2000). Tissue classification with gene expression profiles.. J Comput Biol.

[29] Golub TR, Slonim DK, Tamayo P, Huard C, Gaasenbeek M (1999). Molecular classification of cancer: class discovery and class prediction by gene expression monitoring.. Science.

[30] Barabasi AL, Oltvai ZN (2004). Network biology: understanding the cell's functional organization.. Nat Rev Genet.

[31] Gavin AC, Aloy P, Grandi P, Krause R, Boesche M (2006). Proteome survey reveals modularity of the yeast cell machinery.. Nature.

[32] Tibshirani R, Hastie T, Narasimhan B, Chu G (2002). Diagnosis of multiple cancer types by shrunken centroids of gene expression.. Proc Natl Acad Sci U S A.

[33] Ganter B, Tugendreich S, Pearson CI, Ayanoglu E, Baumhueter S (2005). Development of a large-scale chemogenomics database to improve drug candidate selection and to understand mechanisms of chemical toxicity and action.. J Biotechnol.

[34] Natsoulis G, Pearson CI, Gollub J, B PE, Ferng J (2008). The liver pharmacological and xenobiotic gene response repertoire.. Mol Syst Biol.

[35] Bross IDJ (1958). How to use ridit analysis.. Biometrics.

[36] Donaldson GW (1998). Ridit scores for analysis and interpretation of ordinal pain data.. Eur J Pain.

[37] Lu C, King RD (2009). An investigation into the population abundance distribution of mRNAs, proteins, and metabolites in biological systems.. Bioinformatics.

[38] Pachot A, Blond JL, Mougin B, Miossec P (2004). Peptidylpropyl isomerase B (PPIB): a suitable reference gene for mRNA quantification in peripheral whole blood.. J Biotechnol.

[39] Cai JH, Deng S, Kumpf SW, Lee PA, Zagouras P (2007). Validation of rat reference genes for improved quantitative gene expression analysis using low density arrays.. Biotechniques.

[40] Coussens L, Yang-Feng TL, Liao YC, Chen E, Gray A (1985). Tyrosine kinase receptor with extensive homology to EGF receptor shares chromosomal location with neu oncogene.. Science.

[41] Olayioye MA (2001). Update on HER-2 as a target for cancer therapy: intracellular signaling pathways of ErbB2/HER-2 and family members.. Breast Cancer Res.

[42] Moreau A, Vilarem MJ, Maurel P, Pascussi JM (2008). Xenoreceptors CAR and PXR activation and consequences on lipid metabolism, glucose homeostasis, and inflammatory response.. Mol Pharm.

[43] Adams DH, Ju C, Ramaiah SK, Uetrecht J, Jaeschke H (2010). Mechanisms of immune-mediated liver injury.. Toxicol Sci.

[44] Natsoulis G, El Ghaoui L, Lanckriet GR, Tolley AM, Leroy F (2005). Classification of a large microarray data set: algorithm comparison and analysis of drug signatures.. Genome Res.

[45] Peterson JE (1990). Biliary hyperplasia and carcinogenesis in chronic liver damage induced in rats by phomopsin.. Pathology.

